# Association between the habitual lip and tongue posture, clinical characteristics, and sleep-related problems in infants with Trisomy 21

**DOI:** 10.1590/2317-1782/e20240095en

**Published:** 2025-03-31

**Authors:** Gabriella Marra Guimarães Rezende, Ana Elisa Ribeiro Fernandes, Anna Vitória Mendes Viana Silva, Larissa Melgaço Campos, Yasmim Carvalho Telson, Andréa Rodrigues Motta, Henrique Pretti, Renata Maria Moreira Moraes Furlan

**Affiliations:** 1 Universidade Federal de Minas Gerais – UFMG - Belo Horizonte (MG), Brasil.; 2 Departamento de Pediatria, Faculdade de Medicina, Universidade Federal de Minas Gerais – UFMG - Belo Horizonte (MG), Brasil.; 3 Programa de Pós-graduação em Odontologia, Universidade Federal de Minas Gerais – UFMG - Belo Horizonte (MG), Brasil.; 4 Programa de Pós-graduação em Ciências Fonoaudiológicas, Universidade Federal de Minas Gerais – UFMG - Belo Horizonte (MG), Brasil.; 5 Departamento de Fonoaudiologia, Faculdade de Medicina, Universidade Federal de Minas Gerais – UFMG - Belo Horizonte (MG), Brasil.; 6 Departamento de Dentística Restauradora, Faculdade de Odontologia, Universidade Federal de Minas Gerais – UFMG - Belo Horizonte (MG), Brasil.

**Keywords:** Down Syndrome, Sleep, Muscle Tonus, Sleep Apnea, Obstructive, Stomatognathic System

## Abstract

**Purpose:**

To analyze the association of habitual lip and tongue posture and clinical characteristics with sleep-related problems in infants with Trisomy 21 (T21).

**Methods:**

This cross-sectional observational study with a non-probabilistic sample included 87 infants with T21 with a mean age of 8.8 months. The infants' parents answered the Brief Infant Sleep Questionnaire (BISQ) and questions about signs and symptoms related to obstructive sleep apnea. The habitual lip and tongue posture was obtained by analyzing videos of the infants' faces. Information on personal data and health history was extracted from medical records, and information about feeding and oral habits was obtained by interviewing the parents. Descriptive analysis approached the infants' sleep data and the association between sleep quality, snoring, witnessed respiratory pauses, unusual sleeping positions, restless sleep, and the other variables, using Pearson's chi-square test with a 5% significance level.

**Results:**

Most infants (82.7%) had good sleep quality. Prematurity was associated with witnessed respiratory pauses; unusual sleeping positions were associated with being a female and with the tongue habitually contained in the oral cavity; and restless sleep was associated with choking.

**Conclusion:**

Prematurity, sex, habitual tongue posture, and choking were associated with the aspects of sleep investigated in infants with T21.

## INTRODUCTION

Trisomy 21 (T21), better known as Down syndrome (DS), is a genetic condition resulting from an anomaly in cell division during conception, leading to three chromosome 21s in all or most of an individual's cells^(1)^. People with T21 have physical characteristics in common, such as slanted eyes, rounded faces, smaller hands, and shorter stature^(2)^. They may also have functional impairment and a series of comorbidities, including cardiac malformations^(1)^, visual^(3)^ and auditory^(4)^ changes, gastrointestinal abnormalities^(1)^, obstructive sleep apnea (OSA)^(5)^, recurrent respiratory infections^(4)^, thyroid disorders^(4)^, obesity^(4)^, atlantoaxial dislocation^(4)^, and so on. Early diagnosis and treatment of comorbidities are essential to improve quality of life^(4)^.

Several of these comorbidities, such as global muscle hypotonia, palatine tonsil and adenoid hypertrophy^(6)^, obesity, gastroesophageal reflux disease, hypothyroidism, and congenital heart disease increase this population’s predisposition to sleep disorders^(4)^.

Sleep disorders in children and adolescents are associated with various physical, behavioral, and physiological development problems, posing an additional risk for obesity, endocrine disorders, depression, and immunological and cardiac diseases^(7)^. These disorders are even more detrimental for individuals with T21, who often have these conditions^(7)^.

OSA is the most prevalent sleep disorder in this population, affecting approximately 69 to 79% of children, half of them with moderate to severe apnea^(5)^. Snoring and OSA occur due to total or partial obstruction of air intake during inspiration and are related to hypotonia of the tongue, soft palate, and posterior pharyngeal wall^(6)^. However, sleep is greatly important for these people’s well-being and health^(5,7)^.

Some authors address the relationship between T21 and sleep-disordered breathing^(8)^. A greater understanding of the topic enables professionals who work with this population to propose more targeted and individualized treatments to improve their care.

Hence, this study aimed to analyze the association of habitual lip and tongue posture and clinical characteristics with sleep-related problems in infants with T21. The study hypothesized that these factors are associated with the infants’ sleep quality.

## METHODS

This cross-sectional observational study with a non-probabilistic sample was approved by the Research Ethics Committee of the Federal University of Minas Gerais (UFMG) under evaluation reports no. 4.381.966 and 6.538.851 and CAAE: 37828920.1.0000.5149.

### Participants

The study included 87 infants (42 females and 45 males) with T21, with a mean corrected age of 8.8 months (standard deviation of 6.1 months, minimum age of 1 month, and maximum of 24 months), assisted by the outreach program, “Multidisciplinary approach to orofacial hypotonia and tongue protrusion in babies with Down syndrome”, carried out at UFMG’s School of Dentistry.

The inclusion criteria were infants up to 2 years of corrected age, diagnosed with T21. Infants with other syndromes and/or associated orofacial malformations were excluded.

All infants’ parents/guardians signed an informed consent form.

### Data extraction and variables

The following information on the infants was collected from their medical records in the outreach program: personal data (sex and age) and health history (prematurity, lung disease, heart disease, and hypothyroidism). The corrected age was calculated for infants born prematurely.

### Questionnaires on sleep

A pediatrician certified in sleep medicine collected sleep information through the Brief Infant Sleep Questionnaire (BISQ)^(9,10)^ and questions about signs and symptoms associated with OSA.

Several countries use the BISQ to assess the sleep habits of children aged 0 to 3 years over the previous week. Its 12 questions investigate the child's sleeping habits, sleeping position, average sleep time (day and night), average number of times the child wakes up per night, time the child remains awake during the night, time the child takes to fall asleep, how the child falls asleep and at what time, and whether the parents consider their child's sleep a problem^(10)^. Poor sleep quality is defined as the presence of one of the following criteria: more than three awakenings per night, time awake per night greater than 1 hour, and total sleep time of less than 9 in 24 hours^(9)^.

The authors of the BISQ assessed its test-retest reliability, validated it with known groups by comparing infants with and without sleep-related issues, and compared the BISQ results with those of actigraphy (an objective method of monitoring the sleep/wake cycle) and the parents’ detailed monitoring reports of their child's sleep^(9)^. The instrument’s test-retest measures were strongly correlated, demonstrating good reliability, and correctly classified 85% of infants with and without sleep problems – a better result than that of actigraphy (which correctly classified 65% of infants) and the parents’ detailed reports (81%)^(9)^. Nunes et al. translated the BISQ into Brazilian Portuguese^(10)^. The validation study of the BISQ for the Brazilian population, with 586 parents of infants at 3, 6, 12, and 24 months, found high specificity of the BISQ indicators compared with actigraphy^(11)^.

Questions about signs and symptoms associated with OSA were based on a questionnaire developed by Sanders et al.^(12)^. They use a Likert scale and address the snoring frequency and intensity, whether the child struggles to breathe while sleeping, whether there are respiratory pauses, how often parents need to wake the child with episodes of apnea, sleeping in unusual positions, restless sleep, the frequency of nocturnal awakenings, mouth breathing, whether they have difficulty waking up, and signs of excessive daytime sleepiness (unusual daytime sleepiness, hyperactivity, or restlessness)^(12)^.

### Data collection and habitual tongue and lip posture

The speech-language-hearing team interviewed the parents to obtain data on feeding and oral habits (breastfeeding, use of pacifiers and bottles, choking episodes, anterior food spillage, and thumb-sucking habits).

Forty (46%) of the 87 study participants were randomly selected to analyze their habitual lip and tongue posture through 5-minute videos. The videos focused on the infant's face, positioned in a child seat or on the caregiver’s lap, who was instructed not to interfere with the recordings. Age-appropriate toys were used to distract the infant and capture their habitual lip and tongue posture. The video analysis quantified the time the infant remained in each of the following tongue postures: I) contained in the oral cavity (tongue behind the lower gum line or the lower incisor teeth); II) between the gum lines (tongue over the lower gum line and behind the lower lip); and III) over the lower lip (tongue touching the lower lip)^(13)^. Lip posture was classified as: I) closed (contact between the lower and upper lips throughout the labial rim); II) parted (contact between the upper and lower lips only near the corners of the mouth); or III) open (no contact between the upper and lower lips)^(13)^. The researchers counted the seconds in which the infant remained in each habitual lip and tongue posture, excluding the moments when the infant smiled or vocalized from the analysis. Then, they recorded the infant’s predominant habitual lip and tongue posture.

### Data analysis

The study’s response variables were sleep quality, snoring, respiratory pauses, unusual sleeping position, and restless sleep. Poor sleep quality was defined as follows, using the BISQ criteria: more than three awakenings per night, being awake for more than 1 hour per night, and total sleep time less than 9 in 24 hours.

The explanatory variables were sex; prematurity, lung disease, heart disease, hypothyroidism, breastfeeding, pacifier use, bottle use, choking, anterior food spillage, thumb-sucking habit, and habitual lip and tongue posture.

Data organization and statistical analysis were performed using the STATA 13 program. The data distribution and frequency for categorical variables and measures of central tendency and dispersion for numerical variables were determined to characterize the sample. The response variables were associated with the explanatory variables using Pearson's chi-square test, with a 5% significance level.

## RESULTS

Table 1 presents the 87 infants’ characteristics, of which 45 (51.7%) were males. Most individuals were born at term (mean gestational age of 37.0 weeks, SD of 1.8 weeks, minimum of 31 and maximum of 41 weeks), did not have lung disease or hypothyroidism, but had cardiac changes. Regarding feeding and oral habits, most individuals were exclusively or supplementarily breastfed, used a bottle but not a pacifier, and did not suck their thumbs. More than a quarter of the sample had problems with choking and anterior food spillage. Table 1 also presents the predominant habitual lip and tongue posture of the 40 infants who underwent this analysis. There was a prevalence of open lips, followed by parted and closed lips; and the tongue contained in the oral cavity, followed by over the lips and between the alveolar ridges.

**Table 1:** Characteristics of the sample (n = 87) and habitual lip and tongue posture (n = 40)

Variables	n	%
Prematurity		
Yes	28	32.1
No	59	67.8
Pulmonary disease		
Yes	6	6.9
No	81	93.1
Heart disease		
Yes	53	60.9
No	34	39.1
Hypothyroidism		
Yes	9	10.3
No	74	85.1
Under investigation	4	4.6
Breastfeeding		
Yes	66	77.6
No	19	22.3
Not reported	2	2.3
Pacifier use		
Yes	26	29.8
No	59	67.8
Not reported	2	2.3
Baby bottle use		
Yes	61	70.1
No	24	27.6
Not reported	2	2.3
Choking		
Yes	25	28.7
No	58	66.7
Not reported	4	4.6
Anterior food spillage		
Yes	27	31.0
No	56	64.4
Not reported	4	4.6
Thumb sucking		
Yes	25	28.7
No	60	69.0
Not reported	2	2.3
Habitual lip posture		
Closed	2	5.0
Parted	16	40.0
Open	22	55.0
Habitual tongue posture		
Contained in the oral cavity	20	50.0
Between the alveolar ridges	8	20.0
On the lips	12	30.0

**Caption:** n = absolute frequency; % = relative frequency

Table 2 presents the BISQ results assessing the infants’ sleep. Most individuals slept in the bedroom with their parents, in the supine position, for more than 9 in 24 hours, with less than three nighttime awakenings, lasting less than 1 hour. Most fell asleep while being fed, between 7:00 p.m. and 9:00 p.m. Most parents did not consider their child's sleep a problem.

**Table 2:** Parents' responses to the Brief Infant Sleep Questionnaire (BISQ) concerning their child's sleep (n = 87)

BISQ variables	n	%
Sleep organization		
Crib in a separate room	27	31.0
Crib in the parents’ room	42	48.3
Sleeping on the parents’ bed	12	13.8
Crib in the siblings’ room	4	4.6
Other	2	2.3
Most usual sleeping position		
Prone	18	20.7
On the side	29	33.3
Supine	40	46.0
Mean sleep time		
Less than 9 in 24 hours	3	3.4
9 or more in 24 hours	82	94.3
Not reported	2	2.3
Mean number of times they wake up per night		
More than 3 nighttime awakenings	4	4.6
3 or fewer nighttime awakenings	83	95.4
Time spent awake at night		
More than 1 hour	10	11.5
1 hour or less	71	81.6
Not reported	6	6.9
How they fall asleep		
While eating	41	47.1
Being swayed	18	20.7
On the lap	10	11.5
By themselves in the crib	11	12.6
In bed near the parents	7	8.0
Time when they usually fall asleep at night		
7:00 to 9:00 p.m.	50	58.1
9:01 to 11:00 p.m.	33	38.4
After 11:00 p.m.	3	3.5
Parents consider their child's sleep a problem		
Yes	17	19.5
No	70	80.5

**Caption:** n = absolute frequency; % = relative frequency.

The participants slept for a mean of 576.9 minutes per night – equivalent to 9.6 hours (SD = 115.7 minutes, minimum = 150 minutes, maximum = 780 minutes) – and 220.5 minutes during the day – equivalent to 3.7 hours (SD = 137.7 minutes, minimum = 15 minutes, maximum = 720 minutes). The sample’s mean total sleep was 793.6 minutes, equivalent to 13.2 hours (SD = 139.5 minutes, minimum = 480 minutes, maximum = 1260 minutes). They took a mean of 22.5 minutes to fall asleep (SD = 22.9, minimum = 0, maximum = 120 minutes).

The parents reported the following nocturnal signs occurring more than three times a week (“almost always” and “always”), described in decreasing order of frequency: sleeping in unusual positions, restless sleep, waking up in the middle of the night, snoring, sweating, witnessed apnea, respiratory effort, and need for stimulation to resume breathing. The daytime signs and symptoms that occurred more than three times a week were mouth breathing, moodiness upon waking, excessive daytime sleepiness, difficulty waking up during the day, and hyperactivity (Table 3).

**Table 3:** Parents’ responses to the sleep questionnaire for children with T21 (n = 87)

Variables	Never n (%)	Rarely n (%)	Occasionally n (%)	Almost always n (%)	Always n (%)	Unsure n (%)
How often does your child snore when they do NOT have a cold?	39 (44.8)	10 (11.5)	12 (13.8)	9 (10.3)	17 (19.5)	0
How often can you hear your child snoring from outside the room?	76 (87.4)	5 (5.7)	3 (3.4)	2 (2.3)	1 (1.1)	0
How often does your child struggle to breathe while sleeping?	67 (77.0)	6 (6.9)	7 (8.0)	4 (4.6)	3 (3.4)	0
How often does your child's breathing stop and they suddenly sigh?	51 (58.6)	13 (14.9)	11 (12.6)	5 (5.7)	5 (5.7)	2 (2.3)
When your child is sleeping, how often do you touch them to make them breathe again?	79 (90.8)	1 (1.1)	3 (3.4)	1 (1.1)	2 (2.3)	1 (1.1)
How often does your child sleep in unusual positions?	36 (41.4)	4 (4.6)	7 (8.0)	9 (10.3)	31 (35.6)	0
How often does your child have restless sleep?	33 (37.9)	8 (9.2)	8 (9.2)	3 (3.4)	35 (40.2)	0
How often does your child sweat while sleeping?	44 (50.6)	6 (6.9)	11 (12.6)	4 (4.6)	21 (24.14)	1 (1.1)
How often does your child wake up during the night? (More than other children the same age)	38 (43.7)	9 (10.3)	5 (5.7)	6 (6.9)	29 (33.3)	0
How often does your child have difficulty waking up in the morning, even after getting plenty of sleep?	70 (80.5)	4 (4.6)	2 (2.3)	1 (1.1)	10 (11.5)	0
How often is your child grumpy soon after waking up?	71 (81.6)	0	2 (2.3)	2 (2.3)	11 (12.6)	1 (1.1)
How often does your child tend to breathe through their mouth during the day?	26 (29.9)	8 (9.2)	13 (14.9)	2 (2.3)	37 (42.5)	1 (1.1)
How often is your child unusually sleepy during the day?	68 (78.2)	4 (4.6)	3 (3.4)	2 (2.3)	10 (11.5)	0
How often does your child seem more hyperactive or restless than other same-age children?	76 (87.4)	2 (2.3)	1 (1.1)	1 (1.1)	7 (8.0)	0

**Caption:** Never = it has not occurred in the last 6 months; Rarely = less than one night per week; Occasionally = one to three nights per week; Almost always = four to six nights per week; Always = every night; n = absolute frequency; % = relative frequency.

The BISQ criteria resulted in 15 participants with poor sleep quality, corresponding to 17.2% of the sample – 13 (86.7%) of them had one of the criteria and, two (13.3%) had two criteria; no participant had all three criteria.

Sleep quality was not associated with sex, prematurity, the diseases investigated, breastfeeding, oral habits, or habitual lip and tongue posture (Table 4).

**Table 4:** Association between sample characterization variables and habitual lip and tongue posture and sleep quality

Variables	Sleep quality		p-value
	Good	Poor	
Sex			
Males	35	10	0.203
Females	37	5	
Prematurity			
Yes	22	6	0.476
No	50	9	
Pulmonary disease			
Yes	4	2	0.279
No	68	13	
Heart disease			
Yes	43	10	0.616
No	29	5	
Hypothyroidism			
Yes	8	1	0.544
No	60	14	
Breastfeeding			
Yes	54	12	0.810
No	16	3	
Pacifier use			
Yes	20	6	0.549
No	50	9	
Baby bottle use			
Yes	47	14	0.096
No	23	1	
Choking			
Yes	19	6	0.484
No	50	8	
Anterior food spillage			
Yes	21	6	0.612
No	48	8	
Thumb sucking			
Yes	19	6	0.490
No	51	9	
Habitual lip posture			
Closed	2	0	0.755
Parted	13	3	
Open	19	3	
Habitual tongue posture			
Contained in the oral cavity	17	3	0.071
Between the alveolar ridges	5	3	
On the lips	12	0	

Pearson’s chi-square test. p-value ≤ 0.05 considered significant

Regarding OSA-related signs, prematurity was associated with respiratory pauses witnessed by the parents; being female and having the tongue predominantly contained in the cavity were associated with sleeping in unusual positions; and children whose parents did not report choking had more restless sleep (Table 5).

**Table 5:** Association between sample characterization variables and habitual lip and tongue posture, snoring, witnessed respiratory pause, unusual position, and restless sleep reported by parents

Variables	Snoring		p-value	Respiratory pause		p-value	Unusual position		p-value	Restless sleep		p-value
	Yes	No		Yes	No		Yes	No		Yes	No	
Sex												
Males	16	29	0.345	5	38	0.968	15	30	0.014*	16	29	0.114
Females	11	31		5	37		25	17		22	20	
Prematurity												
Yes	12	16	0.081	6	21	0.041*	11	17	0.388	11	17	0.569
No	22	37		4	54		29	30		27	32	
Pulmonary disease												
Yes	26	55	0.430	0	6	0.354	3	3	0.838	3	3	0.746
No	1	5		10	69		37	44		35	46	
Heart disease												
Yes	14	39	0.245	6	46	0.935	23	30	0.546	23	30	0.947
No	13	21		4	29		17	17		15	19	
Hypothyroidism												
Yes	5	4	0.580	1	8	0.750	5	4	0.594	4	5	0.743
No	32	42		9	63		34	40		33	41	
Breastfeeding												
Yes	22	44	0.306	10	54	0.066	28	38	0.233	29	37	0.887
No	4	15		0	19		11	8		8	11	
Pacifier use												
Yes	4	22	0.103	4	22	0.528	14	12	0.328	13	13	0.424
No	22	37		6	51		25	34		24	35	
Baby bottle use												
Yes	19	42	0.858	6	54	0.355	30	31	0.331	28	33	0.482
No	7	17		4	19		9	15		9	15	
Choking												
Yes	5	20	0.187	1	22	0.223	8	17	0.130	6	19	0.019*
No	20	38		8	50		29	29		30	28	
Anterior food spillage												
Yes	10	17	0.340	4	21	0.350	13	14	0.650	9	18	0.200
No	15	41		5	51		24	32		27	29	
Thumb sucking												
Yes	5	20	0.171	3	22	0.993	12	13	0.800	8	17	0.166
No	21	39		7	51		27	33		29	31	
Habitual lip posture												
Closed	1	1	0.775	1	1	0.394	1	1	0.644	0	2	0.457
Parted	5	11		2	12		9	7		7	9	
Open	9	13		3	19		9	13		10	12	
Habitual tongue posture												
Contained in the oral cavity	8	12	0.713	2	17	0.093	11	9	0.024*	8	12	0.887
Between alveolar ridges	2	6		3	4		6	2		4	4	
On the lips	5	7		1	11		2	10		5	7	

Pearson’s chi-square test

* p-value ≤ 0.05 considered significant

## DISCUSSION

Most infants with T21 in the study sample did not have poor quality sleep according to the BISQ, and most parents did not recognize their children's sleep as a problem. On the other hand, many reported symptoms possibly associated with OSA, such as unusual sleeping positions, restless sleep, snoring, sweating, witnessed apneas, and respiratory effort during sleep. The literature has described a weak correlation between negative parent-reported OSA symptoms and polysomnography (PSG) results, which is the gold standard test for diagnosing OSA. Because OSA is highly prevalent in this population, the American Academy of Pediatrics (AAP) recommends that symptoms such as heavy breathing, snoring, unusual sleeping positions, frequent nighttime awakenings, daytime sleepiness, respiratory pauses, and behavioral problems possibly associated with poor-quality sleep be investigated at least once during the first 6 months of life and at every child follow-up visit. If they have symptoms they must be referred for a sleep disorder assessment. It also recommends that all children with T21 aged 3 to 4 years undergo PSG regardless of symptoms^(14)^.

The prevalence of prematurity in the sample was similar to that described in the literature for children with T21. A retrospective study carried out in Southeastern Brazil between 2012 and 2018 found a prevalence of 28.0% of prematurity in infants with T21, with an approximately 2.4 times greater risk of premature births in this population^(15)^. A study with 1.578 children without T21 aged 2 to 15 years using PSG to diagnose OSA found an association between prematurity and OSA^(16)^. Another study^(17)^ found an almost three times higher frequency of sleep-disordered breathing in premature children aged 8 to 11 years without T21 than in children born at term. In this study’s population, prematurity was associated with witnessed respiratory pauses. However, since the study did not include PSG, it cannot state whether these were central or obstructive pauses. Central apneas are related to immature respiratory control possibly occurring in premature infants, mainly up to 43 weeks of corrected age. The periodic breathing pattern that includes central pauses can occur up to 6 months old^(18)^. Moreover, central apneas progressively decrease with age in individuals with T21, being common in those under 2 years old^(19)^, which is the population of this study.

The prevalence of comorbidities in the study sample was similar to that of children with T21. According to the AAP, approximately 40 to 50% of these children have congenital heart disease and 0.65 to 3% have hypothyroidism^(14)^. None of these comorbidities were associated with poor sleep quality through the BISQ or signs associated with OSA. A study^(20)^ evaluated 152 children with T21 aged 2 to 18 years and likewise found no association between congenital heart disease and hypothyroidism and OSA diagnosed by PSG. As in the present study, it included all congenital heart diseases and hypothyroidism^(20)^. On the other hand, a study^(21)^ with 59 infants with a mean age of 44 days, which used PSG to diagnose OSA, found that the combination of dysphagia and heart disease were strong predictors of OSA in this group of infants^(21)^. The infants in the study were very young, and there is no report as to whether the heart disease had already been corrected.

The presence of breastfeeding was also similar to that reported in the literature. A systematic review found frequencies of breastfed children with T21 between 43 and 100%, regardless of the duration of breastfeeding^(22)^. The present study found no association between breastfeeding and the collected sleep data. It is known that breastfeeding promotes healthy upper airway development and that breast milk provides immunological protection against infections. Therefore, breastfeeding may act as a protective factor for OSA. Children who were breastfed for 2 to 5 months, even when supplemented with formula, had a significantly lower severity of OSA diagnosed by PSG than children who were not breastfed^(23)^. The present sample did not investigate the total duration of breastfeeding.

More than a quarter of our sample reported choking and anterior food spillage. According to the AAP, approximately 31 to 80% of children with T21 have feeding difficulties^(14)^. Anatomical and physiological characteristics, such as coordination deficiencies and neuromotor muscle hypotonia, influence the development of oral motor skills, which can result in feeding problems and swallowing dysfunction^(24)^. This finding agrees with the study by Arslan et al.^(25)^, who observed that tongue and lip hypotonia, inefficient tongue lateralization, and difficulty in oral motor control directly influence swallowing. These difficulties can lead to an increased risk of aspiration and nutritional problems, thus highlighting the importance of early and appropriate interventions to improve these infants’ quality of life. The absence of reports of choking was associated with restless sleep. Choking may be caused by a lack of tongue muscle coordination (leading to poor oral motor control) and reduced tongue tone (making it difficult to eject food or saliva). Infants with these disorders would be expected to have more restless sleep. This may be related to the fact that the data were collected through parental reports. In the age group studied (up to 2 years old), parents may be more concerned with feeding difficulties (which is common in this population) than with sleep-related issues (since, in the present sample, a small percentage had poor-quality sleep).

Regarding sleeping position and location, less than half of the infants adopted the supine position and slept in a crib in their parents' room. The supine position is recommended to prevent sudden infant death syndrome, one of the main causes of death in infants up to 1 year old^(26)^. It is worth noting that these recommendations are for infants under 1 year old, whereas the sample of this study included infants up to 2 years old – for whom there are no specific recommendations for safe sleep. However, infants with T21 may be at even greater risk due to craniofacial characteristics, hypotonia, and delayed motor milestone development, such as rolling over, which occurs on average at 6.5 months^(27)^. A study with children with T21 aged 7 to 16 years showed that only 6.1% chose to sleep in the supine position, suggesting that this preference may be an attempt to optimize airflow entry^(28)^. However, health professionals should advise on sleep and safe sleeping environments for infants under 1 year old.

Participants in this study predominantly had open lip posture, present in 55% of them, followed by parted lips (40%) and closed lips (only 5%). These findings agree with other studies that analyzed lip posture in children with T21 through facial videos^(13,29,30)^. Ferreira et al.^(13)^ evaluated the habitual lip posture of four children with T21 with a mean age of 6.7 months and found a predominance of open lips in three of them. Carlstedt et al.^(29)^ evaluated children with T21 with a mean age of 24 months and observed that the children remained, on average, more than 60% of the time with their lips open. Glatz-Noll and Berg^(30)^ evaluated 24 children with T21 with a mean age of 23 months and found an average duration of closed lips of only 25.6 seconds in 300 seconds of recording. The predominant open-lip posture is a common finding in children with T21 due to hypotonia of the lips and jaw-lifting muscles^(13)^. Open lips are commonly associated with mouth breathing^(31)^, which is also associated with OSA^(32)^. However, the study found no association between lip posture and sleep-related variables in the sample.

This study also found that half of the sample had their tongue contained in the oral cavity, followed by over the lips (in 30% of the infants) and between the alveolar ridges (20%). Ferreira et al.^(13)^ likewise observed that half of their sample of four children contained their tongue in the oral cavity. The other two predominantly positioned their tongue over the lower lip. Carlstedt et al.^(29)^ found a predominance of protruding and inactive tongue (motionless and outside the oral cavity) around 20% of the recording time on average. Glatz-Noll and Berg^(30)^ found an average duration of protruding tongue of only 6.4 seconds in 300 seconds of recording. Although the tongue was generally contained in the oral cavity, contrary to what is expected (the tip of the tongue was touching the incisive papillae), infants kept their tongue on the floor of the mouth, with a slightly enlarged dorsum, which directly impacts the breathing pattern, especially during sleep.

In this study, being a female and habitually containing the tongue in the oral cavity were associated with unusual sleeping positions. The literature reports that children with T21 and OSA avoid the supine position, as it is associated with a greater likelihood of upper airway obstruction during sleep. However, none of these studies describe differences between the sexes^(28,33)^. Regarding the habitual tongue posture, although it was contained in the oral cavity and not anteriorized, it was often found on the oral floor, with the tip low and dorsum high, suggesting hypotonia. Unusual sleep positions may be related to an attempt to widen the airway and defend against glossoptosis. Hence, it is speculated that the habitual anteriorized posture could be a protective factor against obstruction. However, this study cannot state that the infants’ unusual positions are related to sleep-disordered breathing because it did not perform PSG. It is important to mention that none of the infants in the sample had ankyloglossia, which would justify the habitual tongue posture on the oral floor.

The limitations of this study were the absence of PSG and the parent-reported variables regarding sleep, feeding, and oral habits – particularly, pacifier use may have been underreported. The strengths of this study include the analysis of habitual lip and tongue posture through videos, allowing for a more careful assessment of this subjective aspect.

This research innovates by associating orofacial myofunctional assessment with sleep-related problems in infants with T21. Studies on speech-language-hearing assessment or rehabilitation of individuals with T21 are generally directed at intervention in speech articulation and language disorders and global muscle hypotonia without mentioning sleep-disordered breathing. Hence, this study opens new avenues, pointing to aspects that deserve further investigation in future research, assessing participants with PSG.

## CONCLUSION

Most infants with T21 had good sleep quality according to their parents' reports, despite having a high prevalence of signs associated with OSA. Prematurity, sex, habitual tongue posture, and choking were factors associated with the aspects of sleep investigated in this study. Being a female was associated with unusual sleeping positions; containing the tongue in the oral cavity was associated with unusual sleeping positions; and choking was associated with restless sleep.
