# Supplementary material for: Association between the habitual lip and tongue posture, clinical characteristics, and sleep-related problems in infants with Trisomy 21
Source: Codas. 2025 Mar 31;37(3):e20240095. doi: 10.1590/2317-1782/e20240095en (PMC11963880; doi:10.1590/2317-1782/e20240095en)
Supplement: Translation

# Translation: Associação entre postura habitual de lábios e de língua, características clínicas e queixas relacionadas ao sono de lactentes com Trissomia do 21

**DOI:** 10.1590/2317-1782/e20240095pt

**Authors:** Gabriella Marra Guimarães Rezende, Ana Elisa Ribeiro Fernandes, Anna Vitória Mendes Viana Silva, Larissa Melgaço Campos, Yasmim Carvalho Telson, Andréa Rodrigues Motta, Henrique Pretti, Renata Maria Moreira Moraes Furlan

**Affiliations:** 1 Universidade Federal de Minas Gerais – UFMG - Belo Horizonte (MG), Brasil.; 2 Departamento de Pediatria, Faculdade de Medicina, Universidade Federal de Minas Gerais – UFMG - Belo Horizonte (MG), Brasil.; 3 Programa de Pós-graduação em Odontologia, Universidade Federal de Minas Gerais – UFMG - Belo Horizonte (MG), Brasil.; 4 Programa de Pós-graduação em Ciências Fonoaudiológicas, Universidade Federal de Minas Gerais – UFMG - Belo Horizonte (MG), Brasil.; 5 Departamento de Fonoaudiologia, Faculdade de Medicina, Universidade Federal de Minas Gerais – UFMG - Belo Horizonte (MG), Brasil.; 6 Departamento de Dentística Restauradora, Faculdade de Odontologia, Universidade Federal de Minas Gerais – UFMG - Belo Horizonte (MG), Brasil.

**Keywords:** Síndrome de Down, Sono, Tono Muscular, Apneia Obstrutiva do Sono, Sistema Estomatognático

## Abstract

**Objetivo:**

analisar a associação da postura habitual de lábios e de língua e características clínicas com queixas relacionadas ao sono em lactentes com Trissomia do 21 (T21).

**Método:**

trata-se de um estudo observacional transversal, com amostra não probabilística, composta por 87 lactentes com T21, com média de idade de 8,8 meses. Os pais dos lactentes responderam ao Questionário sobre Sono na Criança (BISQ) e a perguntas sobre sinais e sintomas relacionados à apneia obstrutiva do sono. A postura habitual de lábios e língua foi obtida a partir da análise de vídeos da face dos lactentes. Informações referentes a dados pessoais e histórico de saúde foram extraídas dos prontuários e sobre alimentação e hábitos orais foram obtidas por entrevista aos pais. Foi realizada a análise descritiva dos dados referentes ao sono dos lactentes e análise de associação entre qualidade do sono, queixa de ronco, pausas respiratórias presenciadas, adoção de posições incomuns durante o sono e sono agitado e as demais variáveis, por meio do teste Qui-quadrado de Pearson, com nível de significância de 5%.

**Resultados:**

A maioria dos lactentes (82,7%) apresentou boa qualidade do sono. Houve associação entre prematuridade e relato de pausas respiratórias presenciadas; adotar posições incomuns durante o sono apresentou associação com sexo feminino e com a postura habitual da língua contida na cavidade oral; e sono agitado apresentou associação com queixa de engasgo.

**Conclusão:**

prematuridade, sexo, postura habitual de língua e queixas de engasgos foram fatores associados aos aspectos do sono investigados em lactentes com T21.

## INTRODUÇÃO

A Trissomia do 21 (T21), mais conhecida como Síndrome de Down (SD), é uma condição genética resultante de uma anomalia na divisão celular durante a concepção, levando à presença de três cromossomos 21 em todas ou em grande parte das células de um indivíduo^(1)^. Pessoas com T21 possuem características físicas comuns, como olhos oblíquos, rosto arredondado, mãos menores e menor estatura^(2)^. Além disso, pode haver comprometimento funcional e uma série de comorbidades, incluindo más-formações cardíacas^(1)^, alterações visuais^(3)^ e auditivas^(4)^, anormalidades gastrointestinais^(1)^, apneia obstrutiva do sono^(5)^, infecções respiratórias de repetição^(4)^, distúrbios da tireoide^(4)^, obesidade^(4)^, luxação atlantoaxial^(4)^, entre outros. O diagnóstico e tratamento precoces das comorbidades são essenciais para melhorar a qualidade de vida^(4)^.

Várias dessas comorbidades, como hipotonia muscular global, hipertrofia das tonsilas palatinas e adenoide^(6)^, obesidade, doença do refluxo gastroesofágico, hipotireoidismo e cardiopatias congênitas aumentam a predisposição para os distúrbios do sono nesta população^(4)^.

Os distúrbios do sono em crianças e adolescentes estão associados a diversos problemas no desenvolvimento físico, comportamental e fisiológico, representando um risco adicional para obesidade, distúrbios endócrinos, depressão, doenças imunológicas e cardíacas^(7)^. Esses distúrbios são ainda mais prejudiciais para indivíduos com T21, que já enfrentam frequentemente essas condições^(7)^.

Dentre os distúrbios do sono, o mais prevalente nessa população é a apneia obstrutiva do sono (AOS), que afeta cerca de 69 a 79% das crianças, sendo que metade delas apresentam apneia moderada e grave^(5)^. O ronco e a AOS ocorrem devido à obstrução, total ou parcial, da entrada de ar na inspiração, e estão relacionados à hipotonia da língua, palato mole e parede posterior da faringe^(6)^. Ressalta-se a importância do sono para o bem-estar e saúde desses indivíduos^(5,7)^.

Autores abordam a relação da T21 com os distúrbios respiratórios do sono^(8)^. Dessa forma conhecer melhor o tema, possibilitará aos profissionais que atuam com essa população propor tratamentos mais direcionados e individualizados, a fim de melhorar a assistência a esses pacientes.

Considerando os aspectos mencionados, o objetivo do presente estudo foi analisar a associação da postura habitual de lábios e de língua e características clínicas com queixas relacionadas ao sono em lactentes com Trissomia do 21 (T21). A hipótese do estudo é que esses fatores apresentam associação com a qualidade do sono dos lactentes.

## MÉTODO

Este é um estudo de delineamento observacional transversal, com amostra não probabilística, aprovado pelo Comitê de Ética em Pesquisa da Universidade Federal de Minas Gerais (UFMG) sob os pareceres nº 4.381.966 e nº 6.538.851, CAAE: 37828920.1.0000.5149.

### Participantes

Participaram do estudo 87 lactentes com Trissomia do 21, com média de idade corrigida de 8,8 meses (desvio padrão de 6,1 meses, idade mínima de 1 mês e máxima de 24 meses), sendo 42 do sexo feminino e 45 do masculino, atendidos pelo projeto de extensão “Abordagem multidisciplinar da Hipotonia Orofacial e Protrusão lingual em bebês com Síndrome de Down”, realizado na Faculdade de Odontologia da UFMG.

Os critérios de inclusão foram: lactentes com até 2 anos de idade corrigida, com diagnóstico de Trissomia do 21. Foram excluídos os lactentes com outras síndromes e/ou más-formações orofaciais associadas.

Todos os pais ou responsáveis pelos lactentes assinaram o Termo de Consentimento Livre e Esclarecido (TCLE).

### Extração de dados e variáveis

A coleta de informações sobre os lactentes foi realizada por meio dos dados dos prontuários no referido projeto de extensão. As informações extraídas foram: dados pessoais (sexo e idade) e histórico de saúde (prematuridade, doença pulmonar, alteração cardíaca e hipotireoidismo). Foi calculada a idade corrigida para os lactentes que nasceram prematuros.

### Questionários sobre o sono

As informações sobre o sono foram coletadas por uma pediatra certificada na área de Medicina do Sono. Foram utilizados o questionário “Brief Infant Sleep Questionnaire” (BISQ)^(9,10)^ e perguntas sobre sinais e sintomas associados a AOS.

O BISQ é um instrumento utilizado em vários países para avaliar os hábitos de sono, durante a última semana, de crianças na faixa etária de 0 a 3 anos. Ele é composto por 12 questões e investiga a organização da criança para dormir, posição em que dorme, tempo médio de sono (dia e noite), média de quantas vezes acorda por noite, tempo em que a criança permanece acordada durante a noite, tempo que leva para adormecer, como adormece e que horas e se os pais consideram o sono do filho como um problema^(10)^. Qualidade ruim de sono é definida pela presença de um dos seguintes critérios: mais de três despertares por noite, período de permanência acordado por noite maior que uma hora ou tempo total de sono menor que nove em 24 horas^(9)^.

Os autores do BISQ avaliaram a confiabilidade do teste-reteste, realizaram a validação por grupos conhecidos, comparando bebês com e sem queixas relacionadas ao sono, bem como compararam os resultados do BISQ com os da actigrafia, método objetivo de monitoramento do ciclo de sono/vigília, e com os relatos detalhados dos pais quanto ao monitoramento do sono do filho^(9)^. O instrumento apresentou correlação forte entre as medidas do teste-reteste, demonstrando boa confiabilidade, e foi capaz de classificar corretamente 85% dos bebês com e sem queixas de sono, sendo este resultado melhor do que a actigrafia (que classificou corretamente 65% dos bebês) e do que relato detalhado dos pais (81%)^(9)^. A tradução para o Português Brasileiro foi realizada por Nunes et al.^(10)^. O estudo de validação do BISQ para a população brasileira, realizado com 586 pais de bebês aos três, seis, 12 e 24 meses, verificou alta especificidade dos indicadores do BISQ na comparação com a actigrafia^(11)^.

As perguntas sobre sinais e sintomas associados a AOS foram baseadas em um questionário desenvolvido por Sanders e colaboradores^(12)^. Essas perguntas adotaram um formato de escala Likert e abordam frequência e intensidade do ronco, se a criança luta para respirar enquanto dorme, se tem pausa respiratória, a frequência com que os pais precisam acordar o filho com episódios de apneia, sono em posições incomuns, se o sono é agitado, a frequência de despertares noturnos, respiração oral, se há dificuldade ao acordar e sinais de sonolência excessiva diurna (sono incomum durante o dia, hiperatividade ou inquietação)^(12)^.

### Coleta de informações e postura habitual de língua e lábios

Os dados referentes à alimentação e hábitos orais (amamentação, uso de chupeta e mamadeira, presença de engasgo e de escape anterior de alimento e hábito de sucção digital) foram obtidos por entrevista aos pais pela equipe de Fonoaudiologia.

Dos 87 participantes do estudo, 40 (46%) foram selecionados aleatoriamente para análise da postura habitual de lábios e língua, por meio de vídeos de 5 minutos de duração. Os vídeos focaram a face do lactente, posicionado no bebê conforto ou no colo do responsável, o qual foi orientado a não interferir nas gravações. Brinquedos apropriados a cada idade foram oferecidos para manter o lactente distraído para captar a postura habitual de lábios e língua. Na análise dos vídeos, foi quantificado o tempo em que o lactente permaneceu em cada uma das seguintes posturas de língua: I) contida na cavidade oral (língua atrás do rolete gengival inferior ou atrás dos dentes incisivos inferiores); II) entre os roletes gengivais (língua sobre o rolete gengival inferior e atrás do lábio inferior); III) sobre o lábio inferior (língua tocando o lábio inferior)^(13)^. Já a postura de lábios foi classificada como: I) fechados (presença de contato entre os lábios inferior e superior em toda a extensão da rima labial); II) semiabertos (o contato entre os lábios superior e inferior ocorre apenas próximo às comissuras labiais); ou III) abertos (não há contato entre lábio inferior e superior)^(13)^. Foram contabilizados os segundos em que o lactente permaneceu em cada classificação da postura habitual de lábios e de língua, sendo que os momentos em que o lactente sorriu ou vocalizou foram excluídos da análise. Em seguida, foi registrada a postura habitual de lábios e de língua predominantemente adotada pelo lactente.

### Análise de dados

Qualidade do sono, presença de ronco, presença de pausa respiratória, posição incomum ao dormir e presença de sono agitado constituíram as variáveis resposta deste estudo. Foram utilizados os critérios do BISQ para definir uma qualidade ruim de sono: presença de mais de três despertares por noite, período de permanência acordado por noite maior que uma hora ou tempo total de sono menor que nove em 24 horas.

As variáveis explicativas foram: sexo; prematuridade, doença pulmonar, doença cardíaca, hipotireoidismo, amamentação, uso de chupeta, uso de mamadeira, queixa de engasgo, queixa de escape posterior de alimento, hábito de sucção digital e postura habitual de lábios e de língua.

A organização dos dados e a análise estatística foram realizadas utilizando o programa STATA 13. Para caracterização da amostra, foi determinada a distribuição e frequência dos dados para as variáveis categóricas e medidas de tendência central e dispersão para as variáveis numéricas. As variáveis respostas foram associadas às explicativas utilizando-se o Teste Qui-quadrado de Pearson, com nível de significância de 5%.

## RESULTADOS

A Tabela 1 apresenta as características da amostra de 87 lactentes, dos quais 45 (51,7%) eram do sexo masculino. Observou-se que a maioria dos indivíduos nasceu a termo (média da idade gestacional de 37,0 semanas, DP de 1,8 semanas, mínimo de 31 e máximo de 41 semanas), não apresentava doença pulmonar ou hipotireoidismo, mas apresentava alteração cardíaca. Com relação à alimentação e hábitos orais, a maioria dos indivíduos recebeu aleitamento materno, exclusivo ou complementado, fazia uso de mamadeira, mas não de chupeta, e não realizava sucção digital. Mais de um quarto da amostra tinha queixa de engasgos e escape anterior de alimento. A Tabela 1 também apresenta a postura habitual de lábios e de língua predominantemente adotada pelos 40 lactentes que foram submetidos a essa análise. A postura habitual de lábios mais prevalente foi a de lábios abertos, seguida de semiabertos e fechados. A postura de língua mais prevalente foi a contida na cavidade oral, seguida de sobre os lábios e entre os roletes gengivais.

**Tabela 1:** Características da amostra (n=87) e postura habitual de lábios e de língua (n=40)

Variáveis	n	%
Prematuridade		
Sim	28	32.1
Não	59	67,8
Doença pulmonar		
Sim	6	6,9
Não	81	93,1
Doença cardíaca		
Sim	53	60,9
Não	34	39,1
Hipotireoidismo		
Sim	9	10,3
Não	74	85,1
Em investigação	4	4,6
Em amamentação		
Sim	66	77,6
Não	19	22,3
Não informado	2	2,3
Em uso de chupeta		
Sim	26	29,8
Não	59	67,8
Não informado	2	2,3
Em uso de mamadeira		
Sim	61	70,1
Não	24	27,6
Não informado	2	2,3
Queixa de engasgo		
Sim	25	28,7
Não	58	66,7
Não informado	4	4,6
Queixa de escape anterior do alimento		
Sim	27	31,0
Não	56	64,4
Não informado	4	4,6
Hábito de sucção digital		
Sim	25	28,7
Não	60	69,0
Não informado	2	2,3
Postura habitual de lábios		
Fechados	2	5,0
Semiabertos	16	40,0
Abertos	22	55,0
Postura habitual de língua		
Contida na cavidade oral	20	50,0
Entre os roletes gengivais	8	20,0
Sobre os lábios	12	30,0

**Legenda:** n=frequência absoluta; %=frequência relativa

A Tabela 2 apresenta os resultados obtidos com a aplicação do Questionário BISQ para avaliação do sono na primeira infância. A maior parte dos indivíduos dorme no quarto com os pais, na posição supina, por mais de nove em 24 horas, com menos de três despertares noturnos, os quais duram menos de uma hora. A maior parte adormece sendo alimentado, entre 19h00 e 21h00. A maioria dos pais não considera o sono do filho como um problema.

**Tabela 2:** Respostas dos pais sobre o sono do filho obtidas pelo Questionário BISQ (n=87)

Variáveis do BISQ	n	%
Organização para dormir		
Berço em um quarto separado	27	31,0
Berço no quarto dos pais	42	48,3
Na cama dos pais	12	13,8
Berço no quarto com irmãos	4	4,6
Outro	2	2,3
Posição que dorme na maior parte das vezes		
Barriga para baixo	18	20,7
De lado	29	33,3
De costas	40	46,0
Tempo médio de sono		
Menos que 9 horas em 24 horas	3	3,4
Igual ou mais que 9 horas em 24 horas	82	94,3
Não informado	2	2,3
Média de vezes que acorda por noite		
Mais de 3 despertares noturnos	4	4,6
Igual ou menos de 3 despertares noturnos	83	95,4
Tempo que permanece acordado durante a noite		
Maior que uma hora	10	11,5
Menor ou igual a uma hora	71	81,6
Não informado	6	6,9
Como adormece		
Sendo alimentado	41	47,1
Sendo embalado	18	20,7
No colo	10	11,5
Sozinho na sua cama	11	12,6
Na cama perto dos pais	7	8,0
Horário em que normalmente adormece a noite		
Entre 19h00 e 21h00	50	58,1
Entre 21h01 e 23h00	33	38,4
Acima de 23h00	3	3,5
Pais consideram o sono do filho um problema		
Sim	17	19,5
Não	70	80,5

**Legenda:** n=frequência absoluta; %=frequência relativa

A média de sono por noite dos participantes foi de 576,9 minutos, equivalente a 9,6 horas (DP= 115,7 minutos, mínimo=150 minutos, máximo de 780 minutos). Já a média de sono por dia dos participantes foi de 220,5 minutos, equivalente a 3,7 horas (DP=137,7 minutos, mínimo=15 minutos, máximo=720 minutos). A média de sono total da amostra foi de 793,6 minutos, equivalente a 13,2 horas (DP=139,5 minutos, mínimo=480 minutos, máximo=1260 minutos). Já o tempo médio que levam para adormecer foi de 22,5 minutos (DP=22,9, mínimo=0, máximo=120 minutos).

Os sinais noturnos relatados pelos pais, que aconteceram mais de três vezes por semana (“quase sempre” e “sempre”), descritos por ordem decrescente de frequência, foram: dormir em posições incomuns, sono agitado, despertares no meio da noite, ronco, sudorese, apneia presenciada, esforço respiratório e necessidade de ser estimulado para voltar a respirar. Os sinais e sintomas diurnos que aconteceram mais de três vezes por semana foram: respiração oral, mau humor ao acordar, sonolência excessiva diurna, dificuldade de despertar para o dia e hiperatividade (Tabela 3).

**Tabela 3:** Respostas dos pais obtidas pelo Questionário do Sono de Crianças com T21 (n=87)

Variáveis	Nunca n (%)	Raramente n (%)	Ocasional- mente n (%)	Quase sempre n (%)	Sempre n (%)	Não tenho certeza n (%)
Com que frequência seu filho ronca quando NÃO está resfriado?	39 (44,8)	10 (11,5)	12 (13,8)	9 (10,3)	17 (19,5)	0
Quantas vezes você pode ouvir seu filho roncando de fora do quarto?	76 (87,4)	5 (5,7)	3 (3,4)	2 (2,3)	1 (1,1)	0
Quantas vezes seu filho luta para respirar enquanto dorme?	67 (77,0)	6 (6,9)	7 (8,0)	4 (4,6)	3 (3,4)	0
Com que frequência a respiração de seu filho para e de repente ele / ela suspira?	51 (58,6)	13 (14,9)	11 (12,6)	5 (5,7)	5 (5,7)	2 (2,3)
Quando seu filho está dormindo, com que frequência você encosta nele para fazê-lo respirar de novo?	79 (90,8)	1 (1,1)	3 (3,4)	1 (1,1)	2 (2,3)	1 (1,1)
Com que frequência seu filho dorme em posições incomuns?	36 (41,4)	4 (4,6)	7 (8,0)	9 (10,3)	31 (35,6)	0
Com que frequência seu filho tem um sono agitado?	33 (37,9)	8 (9,2)	8 (9,2)	3 (3,4)	35 (40,2)	0
Com que frequência seu filho sua enquanto dormindo?	44 (50,6)	6 (6,9)	11 (12,6)	4 (4,6)	21 (24,14)	1 (1,1)
Com que frequência seu filho acorda durante a noite? (Mais do que outras crianças da mesma idade)	38 (43,7)	9 (10,3)	5 (5,7)	6 (6,9)	29 (33,3)	0
Com que frequência seu filho tem dificuldade de acordar de manhã, mesmo depois de ter dormido bastante?	70 (80,5)	4 (4,6)	2 (2,3)	1 (1,1)	10 (11,5)	0
Com que frequência seu filho fica mal- humorado logo que acorda?	71 (81,6)	0	2 (2,3)	2 (2,3)	11 (12,6)	1 (1,1)
Com que frequência seu filho tende a respirar pela boca durante o dia?	26 (29,9)	8 (9,2)	13 (14,9)	2 (2,3)	37 (42,5)	1 (1,1)
Com que frequência seu filho está com sono incomum durante o dia?	68 (78,2)	4 (4,6)	3 (3,4)	2 (2,3)	10 (11,5)	0
Com que frequência seu filho parece mais hiperativo ou inquieto do que outras crianças com idade semelhante?	76 (87,4)	2 (2,3)	1 (1,1)	1 (1,1)	7 (8,0)	0

**Legenda:** Nunca=não ocorreu nos últimos 6 meses; Raramente=menos de uma noite por semana; Ocasionalmente=uma a três noites por semana; Quase sempre=quatro a seis noites por semana; Sempre=toda noite; n=frequência absoluta; %=frequência relativa

Adotando-se os critérios do BISQ, obteve-se uma frequência de 15 participantes com qualidade ruim de sono, o que corresponde a 17,2% da amostra. Destes 13 (86,7%) tinham um dos critérios e dois (13,3%) tinham dois critérios. Nenhum participante apresentou os três critérios.

A qualidade do sono não apresentou associação com o sexo, a presença de prematuridade, as doenças pesquisadas, o aleitamento materno, os hábitos orais ou a postura habitual de lábios e língua (Tabela 4).

**Tabela 4:** Associação entre variáveis de caracterização da amostra e postura habitual de lábios e língua e a qualidade do sono

Variáveis	Qualidade do sono		Valor de p
	Boa	Ruim	
Sexo			
Masculino	35	10	0,203
Feminino	37	5	
Prematuridade			
Sim	22	6	0,476
Não	50	9	
Doença pulmonar			
Sim	4	2	0,279
Não	68	13	
Doença cardíaca			
Sim	43	10	0,616
Não	29	5	
Hipotireoidismo			
Sim	8	1	0,544
Não	60	14	
Em aleitamento materno			
Sim	54	12	0,810
Não	16	3	
Em uso de chupeta			
Sim	20	6	0,549
Não	50	9	
Em uso de mamadeira			
Sim	47	14	0,096
Não	23	1	
Queixa de engasgo			
Sim	19	6	0,484
Não	50	8	
Queixa de escape anterior do alimento			
Sim	21	6	0,612
Não	48	8	
Hábito de sucção digital			
Sim	19	6	0,490
Não	51	9	
Postura habitual de lábios			
Fechados	2	0	0,755
Semiabertos	13	3	
Abertos	19	3	
Postura habitual de língua			
Contida na cavidade oral	17	3	0,071
Entre os roletes gengivais	5	3	
Sobre os lábios	12	0	

Teste Qui-quadrado de Pearson. Valor de p≤0,05 considerado significante

Em relação aos sinais relacionados à AOS, a prematuridade foi associada a presença de pausas respiratórias presenciada pelos pais, sexo feminino e apresentar a língua predominantemente contida na cavidade foram associados a dormir em posições não usuais e crianças cujos pais não relatavam engasgos apresentavam mais sono agitado (Tabela 5).

**Tabela 5:** Associação entre variáveis de caracterização da amostra e postura habitual de lábios e língua e a presença de ronco, pausa respiratória presenciada, posição incomum e sono agitado informados pelos pais

Variáveis	Presença de ronco		Valor de p	Presença de pausa respiratória		Valor de p	Posição incomum		Valor de p	Sono agitado		Valor de p
	Sim	Não		Sim	Não		Sim	Não		Sim	Não	
Sexo												
Masculino	16	29	0,345	5	38	0,968	15	30	0,014*	16	29	0,114
Feminino	11	31		5	37		25	17		22	20	
Prematuridade												
Sim	12	16	0,081	6	21	0,041*	11	17	0,388	11	17	0,569
Não	22	37		4	54		29	30		27	32	
Doença pulmonar												
Sim	26	55	0,430	0	6	0,354	3	3	0,838	3	3	0,746
Não	1	5		10	69		37	44		35	46	
Doença cardíaca												
Sim	14	39	0,245	6	46	0,935	23	30	0,546	23	30	0,947
Não	13	21		4	29		17	17		15	19	
Hipotireoidismo												
Sim	5	4	0,580	1	8	0,750	5	4	0,594	4	5	0,743
Não	32	42		9	63		34	40		33	41	
Em aleitamento materno												
Sim	22	44	0,306	10	54	0,066	28	38	0,233	29	37	0,887
Não	4	15		0	19		11	8		8	11	
Em uso de chupeta												
Sim	4	22	0,103	4	22	0,528	14	12	0,328	13	13	0,424
Não	22	37		6	51		25	34		24	35	
Em uso de mamadeira												
Sim	19	42	0,858	6	54	0,355	30	31	0,331	28	33	0,482
Não	7	17		4	19		9	15		9	15	
Queixa de engasgo												
Sim	5	20	0,187	1	22	0,223	8	17	0,130	6	19	0,019*
Não	20	38		8	50		29	29		30	28	
Queixa de escape anterior do alimento												
Sim	10	17	0,340	4	21	0,350	13	14	0,650	9	18	0,200
Não	15	41		5	51		24	32		27	29	
Hábito de sucção digital												
Sim	5	20	0,171	3	22	0,993	12	13	0,800	8	17	0,166
Não	21	39		7	51		27	33		29	31	
Postura habitual de lábios												
Fechados	1	1	0,775	1	1	0,394	1	1	0,644	0	2	0,457
Semiabertos	5	11		2	12		9	7		7	9	
Abertos	9	13		3	19		9	13		10	12	
Postura habitual de língua												
Contida na cavidade oral	8	12	0,713	2	17	0,093	11	9	0,024*	8	12	0,887
Entre os roletes gengivais	2	6		3	4		6	2		4	4	
Sobre os lábios	5	7		1	11		2	10		5	7	

Teste Qui-quadrado de Pearson

* Valor de p≤0,05 considerado significante

## DISCUSSÃO

Na amostra investigada, a maioria dos lactentes com T21 não apresentou sono de má qualidade pelo BISQ e a maioria dos pais não reconheceu o sono dos filhos como um problema. Por outro lado, muitos deles relataram sintomas que podem estar associados à AOS, como posições incomuns para dormir, sono agitado, ronco, sudorese, apneias presenciadas, esforço respiratório durante o sono. É descrita na literatura, uma fraca correlação entre o relato negativo dos sintomas de AOS pelos pais e os resultados da polissonografia, que é o exame padrão ouro para o diagnóstico de AOS. Pelo fato de a AOS ser muito prevalente nessa população, a Academia Americana de Pediatria (AAP) recomenda que, pelo menos uma vez durante os primeiros 6 meses de vida e em todas as consultas de puericultura, sejam investigados sintomas como respiração pesada, ronco, posições incomuns para dormir, despertares noturnos frequentes, sonolência diurna, pausas respiratórias e problemas de comportamento que possam estar associados a sono de má qualidade. Caso os sintomas estejam presentes, a criança precisa ser encaminhada para avaliação de um possível distúrbio do sono. Entre 3 e 4 anos de idade, a recomendação é que todas as crianças com T21 realizem a polissonografia independente dos sintomas^(14)^.

A prevalência da prematuridade na amostra foi semelhante à descrita na literatura para a população de crianças com T21. Um estudo retrospectivo realizado na região Sudeste do Brasil, entre 2012 e 2018, encontrou uma prevalência de 28,0% de prematuridade em lactentes com T21, com um risco de aproximadamente 2,4 vezes maior de partos prematuros nessa população^(15)^. Um estudo com 1.578 crianças, sem T21, entre 2 e 15 anos, que utilizou polissonografia (PSG) para o diagnóstico de AOS encontrou associação entre prematuridade e AOS^(16)^. Outro estudo^(17)^ encontrou frequência quase três vezes maior de distúrbios respiratórios do sono em crianças de 8 a 11 anos, sem T21, nascidas prematuras em comparação com crianças nascidas a termo. Na população deste estudo, a prematuridade foi associada a queixa de pausas respiratórias presenciadas, mas como o estudo não incluiu a PSG, não se pode afirmar se se trata de pausas centrais ou obstrutivas. Apneias centrais estão relacionadas à imaturidade do controle respiratório que pode ocorrer no lactente prematuro principalmente até 43 semanas de idade corrigida. O padrão de respiração periódica que inclui pausas centrais pode ocorrer até 6 meses de idade^(18)^. Além disso, na população de indivíduos com T21 apneias centrais diminuem progressivamente com a idade, sendo comum em indivíduos menores que 2 anos de idade^(19)^, que é a população deste estudo.

A amostra do estudo apresenta prevalências de comorbidades semelhantes à da população de crianças com T21. Segundo a AAP, cerca de 40 a 50% dessas crianças apresentam cardiopatias congênitas e 0,65 a 3% apresentam hipotireoidismo^(14)^. Nenhuma dessas comorbidades foram associadas ao sono de má-qualidade através do BISQ ou sinais associados a AOS. Um estudo^(20)^ avaliou 152 crianças com T21 entre 2 e 18 anos e também não encontrou associação entre cardiopatia congênita e hipotireoidismo e AOS diagnosticada pela PSG. Da mesma forma que no presente estudo, qualquer cardiopatia congênita foi incluída, assim como o hipotireoidismo^(20)^. Por outro lado, um estudo^(21)^ realizado com 59 lactentes, com média de idade de 44 dias, que utilizou a PSG para diagnosticar AOS, encontrou que a combinação de disfagia e cardiopatia foram fortes preditores de AOS nesse grupo de lactentes^(21)^. Os lactentes no referido estudo eram muitos novos e não há relato se a cardiopatia já havia sido corrigida.

A presença do aleitamento materno também foi semelhante à relatada na literatura. Uma revisão sistemática encontrou frequências de crianças com T21 amamentadas entre 43 e 100%, independentemente da duração da amamentação^(22)^. No presente estudo não houve associação entre amamentação e os dados coletados sobre sono. Sabe-se que a amamentação promove o desenvolvimento saudável das vias aéreas superiores e que o leite materno confere proteção imunológica contra infecções. Por isso, acredita-se que a amamentação atue como fator protetor para AOS. Crianças que foram amamentadas por 2 a 5 meses, mesmo que complementado com fórmula, apresentaram gravidade significativamente reduzida da AOS diagnosticada pela PSG do que crianças que não foram amamentadas^(23)^. Na presente amostra não foi investigado o tempo total de aleitamento materno.

Mais de um quarto da nossa amostra relatou queixa de engasgos e escape anterior de alimento. Segundo a AAP, cerca de 31 a 80% das crianças com T21 apresentam dificuldades alimentares^(14)^. Características anatômicas e fisiológicas, como deficiências de coordenação e hipotonia muscular neuromotora, influenciam o desenvolvimento de habilidades motoras orais, o que pode resultar em problemas de alimentação e disfunção na deglutição^(24)^. Tal achado concorda com o estudo de Arslan et al.^(25)^ que observaram que a hipotonia de língua e de lábios, a lateralização ineficiente da língua e a dificuldade no controle motor oral influenciam diretamente na deglutição. Essas dificuldades podem levar ao aumento do risco de aspiração e problemas de nutrição, destacando, assim, a importância de intervenções precoces e adequadas para melhorar a qualidade de vida desses lactentes. A ausência de relato de engasgo foi associada a sono agitado. Os engasgos podem ser ocasionados por falta de coordenação muscular da língua, o que leva a um pobre controle motor oral, bem como, por um tônus de língua reduzido, o que dificulta a ejeção de alimento ou de saliva. O esperado seria que os lactentes com essas alterações tivessem o sono mais agitado. Isso pode estar relacionado ao fato de o dado ter sido coletado por meio de relato dos pais. Na faixa etária estudada (até dois anos idade), os pais podem estar mais preocupados com as dificuldades alimentares (que é comum nessa população) do que com as questões relacionadas ao sono (uma vez que, na presente amostra, um percentual pequeno apresentou sono de má-qualidade).

Quanto à posição e ao local de dormir, menos da metade dos lactentes adotavam a posição supina e dormiam em berço no quarto dos pais. A posição supina é a recomendada para evitar a síndrome da morte súbita, uma das principais causas de morte em lactentes de até um ano de idade^(26)^. Vale ressaltar que essas recomendações são para lactentes menores de 1 ano e a amostra deste estudo incluiu até 2 anos de idade. Não existe recomendação específica de sono seguro para esta faixa etária. Porém, lactentes com T21 podem apresentar risco ainda maior devido às características craniofaciais, à hipotonia e ao atraso do desenvolvimento de marcos motores, como o rolar, que acontece em média aos 6,5 meses^(27)^. Um estudo com crianças com T21, de 7 a 16 anos, mostrou que apenas 6,1% escolhiam dormir em supino, sugerindo que esta preferência pode estar relacionada à tentativa de otimizar a entrada de fluxo de ar^(28)^. Porém, em se tratando de lactentes menores de 1 ano, vale ressaltar a importância dos profissionais de saúde aconselharem sobre sono e ambientes seguros para dormir.

A postura labial predominante, verificada nesta pesquisa, foi de lábios abertos, presente em 55% da amostra, seguida de lábios semiabertos (40%) e fechados (apenas 5%). Estes achados concordam com outros estudos que também avaliaram a postura labial em crianças com T21, por meio da análise de vídeos da face^(13,29,30)^. Ferreira et al.^(13)^ avaliaram a postura habitual de lábios de quatro crianças com T21, com média de idade de 6,7 meses, e encontraram predominância de lábios abertos em três delas. Carlstedt et al.^(29)^ avaliaram crianças com T21, com média de idade de 24 meses, e observaram que as crianças permaneceram, em média, mais de 60% do tempo com os lábios abertos. Glatz-Noll e Berg^(30)^ avaliaram 24 crianças com T21, com média de idade de 23 meses, e verificaram duração média de lábios fechados de apenas 25,6 segundos em 300 segundos de gravação. A postura predominante de lábios abertos é um achado comum em crianças com T21 devido à hipotonia dos lábios e da musculatura levantadora da mandíbula^(13)^. Os lábios abertos estão comumente associados à presença da respiração oral^(31)^, que também está associada a AOS^(32)^. Porém, na amostra estudada, não se verificou associação entre a postura de lábios com variáveis relacionadas ao sono.

Com relação à postura de língua, nesta pesquisa, verificou-se que metade da amostra apresentou língua contida na cavidade oral, seguida de sobre os lábios, em 30% dos lactentes, e entre os roletes gengivais (20%). Ferreira et al.^(13)^ também verificaram, em sua amostra de quatro crianças, que metade delas adotavam postura de língua contida na cavidade oral. As outras duas apresentaram predominância de língua sobre o lábio inferior. Carlstedt et al.^(29)^ encontraram predominância de língua protrusa e inativa (imóvel e fora da cavidade oral) em torno de 20%, em média, do tempo de gravação. Glatz-Noll e Berg^(30)^ verificaram duração média de língua protrusa de apenas 6,4 segundos em 300 segundos de gravação. Apesar de a língua estar geralmente contida na cavidade oral, ao contrário do esperado (o ápice da língua encostado nas papilas incisivas), os lactentes mantinham a língua contida em assoalho da boca, com dorso levemente aumentado, o que impacta diretamente no modo respiratório principalmente no sono.

Nesta pesquisa, sexo feminino e a postura habitual de língua contida na cavidade oral foram associados a adotar posições incomuns durante o sono. Há relatos na literatura de que crianças com T21 e AOS evitam a posição supina, pois está associada à maior probabilidade de obstrução da via aérea superior durante o sono. Mas nenhum desses estudos descrevem diferenças entre os sexos^(28,33)^. Em relação à postura habitual de língua, apesar de não estar anteriorizada e estar contida na cavidade oral, encontrava-se frequentemente em assoalho oral, com a ponta baixa e dorso alto, sugerindo hipotonia. Posição incomum no sono pode estar relacionada à tentativa de ampliação da via aérea e defesa contra a glossoptose. Nesse sentido, especula-se que a postura habitual mais anteriorizada poderia ser um fator de proteção contra a obstrução. No entanto, por meio deste estudo, não se pode afirmar que as posições incomuns adotadas pelos lactentes da pesquisa estão relacionadas a um distúrbio respiratório do sono, já que não foi realizada a PSG. É importante mencionar que nenhum dos lactentes da amostra apresentava anquiloglossia, que justificasse a adoção de uma postura habitual em assoalho oral.

Constituíram limitações da presente pesquisa: a ausência do exame de PSG e o fato de as variáveis relacionadas ao sono, à alimentação e aos hábitos orais terem sido obtidas por meio do relato dos pais. Especialmente hábitos de sucção de chupeta podem ter sido sub-relatados. Como pontos fortes desta pesquisa, tem-se a análise da postura habitual de lábios e de língua por meio dos vídeos, o que possibilitou uma avaliação mais criteriosa, visto que se trata de um aspecto subjetivo.

Esta pesquisa inova ao associar aspectos da avaliação miofuncional orofacial com queixas relacionadas ao sono de lactentes com T21. Os estudos sobre avaliação ou reabilitação fonoaudiológica de pessoas com T21 estão geralmente direcionados para a intervenção dos distúrbios fonoarticulatórios, de linguagem e da hipotonia muscular global, sem menção aos distúrbios respiratórios do sono. Nessa perspectiva, este estudo abre caminhos, apontando para os aspectos que merecem maiores investigações em futuras pesquisas que incluam a polissonografia na avaliação dos participantes.

## CONCLUSÃO

A maioria dos lactentes com T21 apresentou boa qualidade do sono pelo relato dos pais, apesar de apresentarem alta prevalência de sinais associados a AOS. Prematuridade, sexo, postura habitual de língua e queixas de engasgos foram fatores associados aos aspectos do sono investigados nesta pesquisa, sendo que houve associação entre sexo feminino e adoção de posições incomuns durante o sono, entre apresentar postura de língua contida em cavidade oral e adoção de posições incomuns durante o sono e entre apresentar queixa de engasgos e ter o sono agitado.
